# Environmental Metal Exposure and Brain-Derived Neurotrophic Factor (BDNF): A Systematic Review of Human and Experimental Evidence

**DOI:** 10.3390/jox16020059

**Published:** 2026-04-02

**Authors:** Maria-Nefeli Georgaki, Despoina Ioannou, Elpis Chochliourou, Kanellos Skourtsidis, Theodora Papamitsou, Dimosthenis Sarigiannis

**Affiliations:** 1 Department of Environmental Engineering, Aristotle University of Thessaloniki, 54124 Thessaloniki, Greece; sarigiannis@eie.gr; 2 HERACLES Environmental Health Research Group, Center for Research and Technology Hellas (CERTH), 57001 Thessaloniki, Greece; 3 Department of Histology and Embryology, School of Medicine, Aristotle University of Thessaloniki, 54124 Thessaloniki, Greece; kskour@auth.gr (K.S.); thpapami@auth.gr (T.P.); 4 Intensive Care Unit, 3rd Pediaric Department, Aristotle University of Thessaloniki, Hippokration General Hospital Thessaloniki, 54124 Thessaloniki, Greece

**Keywords:** brain-derived neurotrophic factor (BDNF), heavy metals, environmental exposure, neurotoxicity, neuroinflammation, neurobehavioral outcomes

## Abstract

Background: Brain-derived neurotrophic factor (BDNF) is central to synaptic plasticity and neurodevelopment. Toxic metal exposure is linked to oxidative stress and neuroinflammation, yet its effects on BDNF signaling remain unclear. Objectives: To systematically synthesize evidence from human and experimental studies on the association between environmental or occupational metal exposure and BDNF alterations, and to highlight research gaps with an emphasis on hexavalent chromium (Cr(VI)). Methods: PubMed, Scopus, and ScienceDirect were searched following PRISMA guidelines. Eligible studies included human observational research and animal models reporting quantitative associations between metal exposure (biomarkers/environmental measures) and BDNF outcomes (protein or gene expression). Data were extracted on exposure assessment, BDNF measurement, and neurobehavioral outcomes. Study quality was assessed using NOS (human studies) and SciRAP (experimental studies). Results: Nineteen studies were included. Across metals such as Pb, Hg, Cd, As, Mn, and mixtures, exposure was associated with altered BDNF levels in blood or brain tissue, often alongside oxidative stress markers, inflammatory changes, and cognitive or behavioral impairment in animal models. Most human studies reported decreased circulating BDNF with higher exposure, while experimental evidence suggested context-dependent regulation across exposure windows and brain regions. Conclusions: The available evidence supports a biologically plausible link between metal exposure and BDNF dysregulation. No eligible studies evaluated BDNF in relation to Cr(VI), indicating a major research gap. Future studies should integrate neurotrophic biomarkers with exposome-oriented designs to clarify chromium-related neurotoxicity and support Adverse Outcome Pathway (AOP)-informed frameworks.

## 1. Introduction

Brain-derived neurotrophic factor (BDNF) is among the most extensively studied neurotrophins and plays a central role in neuronal survival, synaptic plasticity, neurogenesis, and cognitive functioning [1]. By binding to and activating the tropomyosin receptor kinase B (TrkB), BDNF supports long-term potentiation, regulates neurotransmission within glutamatergic and GABAergic circuits, and contributes to stress resilience and emotional regulation. Reduced BDNF availability has been associated with impaired neuroplasticity and altered brain network connectivity and has been implicated in several neuropsychiatric and neurocognitive conditions, including major depressive disorder, bipolar disorder, anxiety disorders, and age-related cognitive decline [2]. Given its functional relevance across multiple domains of brain health, BDNF is increasingly considered a sensitive biomarker reflecting neurobiological stress and environmentally driven adversity.

Exposure to toxic environmental metals including arsenic (As), lead (Pb), cadmium (Cd), mercury (Hg), and manganese (Mn) represent a major and persistent public health challenge worldwide [3]. These metals can accumulate in biological tissues and exert neurotoxic effects through converging mechanisms, such as the generation of reactive oxygen species (ROS), the disruption of mitochondrial function, interference with calcium homeostasis and neurotransmitter systems, and the activation of neuroinflammatory pathways [4]. In both humans and experimental models, metal exposure has been linked to neurodevelopmental disturbances, cognitive impairment, mood-related symptoms, and neurodegenerative processes. While human biomonitoring initiatives like HBM4EU emphasize the need for integrative biomarkers of effect, research has historically focused on conventional toxicological endpoints. However, BDNF has emerged as a promising multi-level indicator, measurable through circulating protein, gene expression, and epigenetic modifications, offering insight into neurobiological vulnerability preceding clinical disease [5,6].

Accumulating evidence suggests that metal-induced oxidative stress and inflammatory mediators suppress BDNF synthesis and interfere with TrkB signaling [7]. In addition, epigenetic regulation such as DNA methylation at BDNF promoter regions may represent a stable mechanism through which chronic metal exposure shapes long-term neurotrophic and behavioral outcomes. While epidemiological and experimental models have documented altered BDNF levels following exposure to As, Cd, Hg, Pb, and Mn, the literature remains fragmented, often restricted to single-metal paradigms that limit cross-metal comparisons and integrated mechanistic interpretations [5,8].

Hexavalent chromium [Cr(VI)] is a widely recognized environmental contaminant with well-established carcinogenic, immunotoxin, and respiratory effects [9,10,11]. Although these effects are well-documented, emerging evidence indicates that Cr(VI) can cross biological barriers and accumulate in brain regions essential for learning and memory, such as the hippocampus [12]. Mechanistically, Cr(VI) induces mitochondrial dysfunction, excessive ROS production, lipid peroxidation, and the activation of apoptotic signaling, and it indicates broad metabolic reprogramming in neural cells leading to neuronal and glial injury [13,14]. Despite this strong mechanistic evidence, no published studies to date have directly examined whether Cr(VI) exposure alters BDNF levels, TrkB signaling, or related neurotrophic mechanisms [5,15]. This represents a critical knowledge gap, given that key processes disrupted by Cr(VI) including redox imbalance, mitochondrial instability, inflammatory signaling, and calcium dysregulation are known regulators of BDNF expression and neurotrophic-mediated plasticity [16]. Furthermore, several metals with overlapping mechanistic profiles (e.g., As, Cd, Pb, Hg) have already been associated with BDNF alterations in humans and experimental systems, suggesting that Cr(VI) may exert comparable or potentially stronger neurotrophic effects [17]. However, the neurotrophic dimension of chromium neurotoxicity remains essentially unexplored. Traditional toxicological frameworks often rely on isolated endpoints, failing to capture the complex, multi-level neurobiological responses that evolve from molecular to functional stages [18]. Because Cr(VI) influences diverse biological layers, there is a critical need for multi-omics and exposome-oriented research [19]. In this framework, neurotrophic biomarkers like BDNF could serve as sensitive indicators of early disruption, linking environmental stressors to downstream cognitive outcomes. However, the current lack of systematic evidence prevents the validation of BDNF as a suitable biomarker in chromium-exposed populations [20]. A comprehensive synthesis is therefore needed to clarify the consistency and biological relevance of reported associations and to identify priorities for future research, as no eligible studies have evaluated BDNF or related neurotrophic endpoints following Cr(VI) exposure.

The present systematic review addresses these gaps by synthesizing evidence from human and experimental studies investigating associations between metal exposure and BDNF-related outcomes. By mapping the strength, consistency, and mechanistic plausibility of reported findings, this review aims to: 1. identify metals that have demonstrated neurotrophic effects through changes in circulating BDNF, brain expression, or epigenetic regulation; 2. evaluate mechanistic pathways linking metal-induced oxidative, inflammatory, and metabolic disruption to altered neurotrophic signaling; 3. highlight the complete absence of evidence for Cr(VI) and provide a rationale for targeted chromium-focused research integrating neurotrophic biomarkers with exposome-based frameworks; 4. propose research directions supporting the development of AOP-informed neurotoxicity models relevant to chromium exposure [21,22]. Ultimately, this review seeks to clarify the role of BDNF as a biomarker of effect in metal-related neurotoxicity and to emphasize the urgent need for mechanistic and epidemiological studies addressing chromium-associated neurotrophic disruption or other related heavy metals.

## 2. Materials and Methods

### 2.1. Study Design

This systematic review was conducted in accordance with the Preferred Reporting Items for Systematic Reviews and Meta-Analyses (PRISMA 2020) guidelines. The review protocol was developed a priori and structured using the PECO framework to define eligibility criteria, guide database searches, support transparent study selection, and ensure reproducible data extraction [23]. Both human epidemiological studies and experimental animal studies were included. Furthermore, mechanistic in vitro studies using primary neuronal cultures were included as supportive evidence to bridge environmental exposure and molecular neurobiological outcomes. Purely biochemical assays or studies using non-neuronal immortalized cell lines were excluded to maintain a translational focus of brain-derived neurotrophic factors (BDNFs) as a neurotrophic biomarker bridging environmental exposure and neurobiological outcomes. In vitro studies were considered as supportive mechanistic evidence when directly assessing BDNF-related pathways under metal exposure.

This systematic review was based exclusively on the published literature and did not require ethical approval. Although the protocol was not registered in PROSPERO, all methodological steps were predefined and conducted in accordance with PRISMA 2020 standards. To ensure a structured and transparent study selection process, eligibility criteria were defined using the PECO (Population, Exposure, Comparator, Outcome) framework, as follows:

Population (P): Human participants of any age, sex, nationality, or health status exposed to environmental or occupational metals, and experimental animals (e.g., rodents, zebrafish, or other vertebrate models) exposed to metals under controlled laboratory conditions.

Exposure (E): Exposure to at least one environmental or occupational metal or metalloid, including arsenic (As), lead (Pb), cadmium (Cd), mercury (Hg), manganese (Mn), nickel (Ni), aluminum (Al), chromium (total Cr or hexavalent chromium [Cr(VI)]), or metal mixtures. Exposure assessment included biological matrices (blood, serum, plasma, urine, hair, nails, or tissues), environmental measurements (water, soil, air), or controlled dosing in experimental models.

Comparator (C): Reference populations or animals with lower or no metal exposure, sham-exposed controls, or baseline exposure groups.

Outcome (O): Quantitative BDNF-related outcomes, including circulating protein levels (serum or plasma BDNF), brain tissue BDNF expression (mRNA or protein), epigenetic regulation of the BDNF gene (e.g., DNA methylation), TrkB receptor expression or signaling, or closely related neurotrophic indicators when clearly linked to BDNF pathways.

Only studies reporting quantitative associations between metal exposure and at least one BDNF-related endpoint were considered eligible.

### 2.2. Information Sources and Search Strategy

A comprehensive literature search was conducted in PubMed/MEDLINE, Scopus, and ScienceDirect, covering all available years up to the final search date (January 2026). Searches were restricted to articles published in English. Reference lists of included studies and relevant reviews were manually screened to identify additional eligible publications. The search strategy combined controlled vocabulary terms and free-text keywords related to neurotrophic factors, metals, and neurotoxicity. The core search syntax was structured as follows: (“BDNF” OR “brain-derived neurotrophic factor”) AND (“metal” OR “heavy metal” OR “arsenic” OR “lead” OR “cadmium” OR “mercury” OR “manganese” OR “nickel” OR “aluminium” OR “chromium” OR “chromium VI” OR “Cr(VI)”) AND (“exposure” OR “toxicity” OR “neurotoxicity” OR “biomarker” OR “epigenetic”). Database-specific adaptations of the search strategy were applied as appropriate (Table S1).

### 2.3. Study Selection Process and Data Extraction

All records retrieved from database searches were imported into reference management software, and duplicates were removed both automatically and manually. Study selection followed a two-stage screening process. First, two reviewers independently screened titles and abstracts to exclude studies that clearly did not meet eligibility criteria. Studies were excluded at this stage if they did not assess metal exposure, did not evaluate BDNF-related outcomes, or represented reviews, editorials, conference abstracts, or cell-line-only investigations lacking primary neuronal or in vivo validation. Second, full-text versions of potentially eligible studies were retrieved and assessed independently by two reviewers. Discrepancies were resolved through discussion, and when consensus could not be reached, a third reviewer served as an arbitrator. The overall selection process is summarized using a PRISMA 2020 flow diagram, documenting records identified, duplicates removed, studies screened, full-text articles assessed, and final inclusions with reasons for exclusion.

Data extraction was performed using a standardized form developed a priori to ensure consistency and reproducibility. Extracted information included study identification (authors, year, country), study design (human observational or experimental), population or species characteristics (age, sex, population group or animal strain and developmental stage), and sample size. Exposure-related data included the metal(s) assessed, type of exposure (environmental, occupational, or experimental), exposure matrix (biological or environmental), dose or concentration, and duration of exposure. BDNF-related outcomes included the type of measure (protein, mRNA, epigenetic modification, or TrkB signaling), biological matrix or brain region, and analytical method (e.g., ELISA, Western blot, qPCR, methylation assays). Reported results included the direction and magnitude of associations, statistical significance, and effect estimates where available. Mechanistic findings, particularly those related to oxidative stress, mitochondrial dysfunction, neuroinflammation, calcium dysregulation, or metabolic alterations, were extracted when reported. Behavioral or cognitive outcomes and additional biomarkers were recorded to support integrative interpretation.

### 2.4. Quality Assessment and Risk of Bias

Quality assessment was conducted independently by two reviewers using tools appropriate to study design. Human observational studies were evaluated using the Newcastle–Ottawa Scale (NOS), assessing participant selection, comparability of exposed and non-exposed groups, outcome or exposure assessment, and control of key confounders (e.g., age, sex, smoking status, body mass index, and socioeconomic factors). Based on NOS criteria, studies were classified as good, fair, or poor quality [24]. Experimental animal studies and supportive in vitro investigations were appraised using the SciRAP criteria, focusing on exposure characterization, outcome assessment, transparency of study design (including randomization and blinding where applicable), and statistical reporting quality [25]. Overall study reliability was categorized as high, medium, or low. Disagreements in quality assessment were resolved through discussion and consensus.

## 3. Results

### 3.1. Study Selection

**Figure 1:**
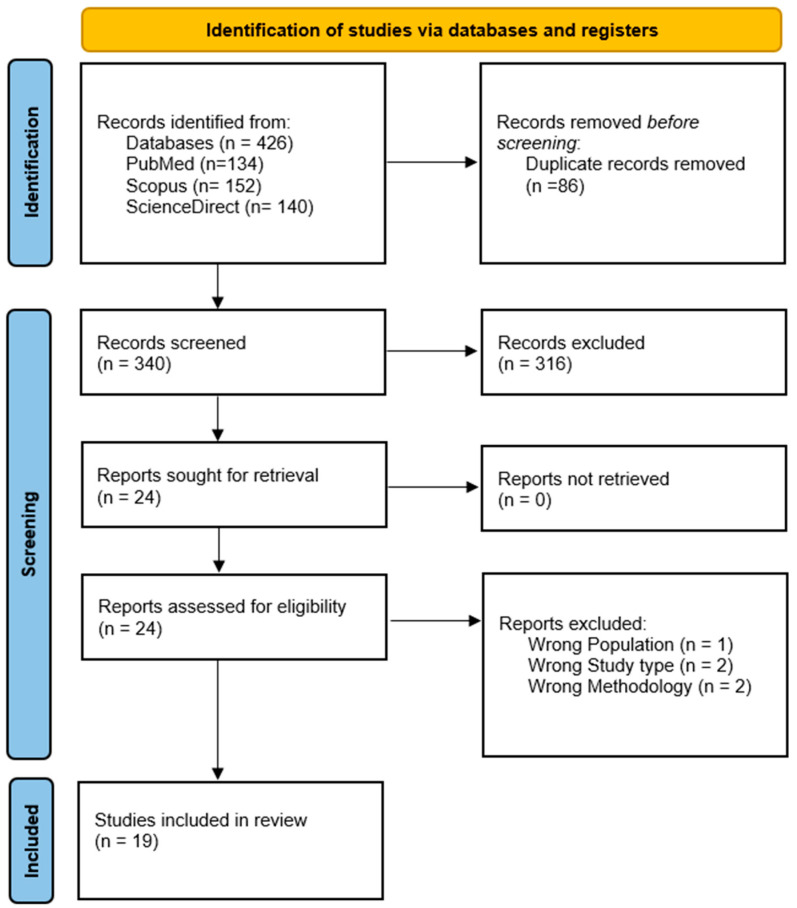
Study selection process illustrated via PRISMA 2020.

The initial database search across PubMed/MEDLINE, Scopus, and ScienceDirect identified a total of 426 records. After the removal of 86 duplicates, 340 unique records were screened by title and abstract. During this initial screening, 316 records were excluded for the following reasons: 184 records did not evaluate any of the specified environmental or occupational metal exposures. Also, 92 records lacked a quantitative BDNF-related endpoint or relevant neurotrophic signaling data and 40 records were non-original publications, such as systematic reviews, meta-analyses, editorials, or conference abstracts. The remaining 24 articles underwent rigorous full-text assessment. Of these, five studies were excluded: one for targeting an ineligible population, two for using an incorrect study design (purely descriptive without a control group), and two for failing to meet quantitative methodology standards. This resulted in the final inclusion of 19 studies. A PRISMA 2020 flow diagram summarizes the study identification, screening, and inclusion process (Figure 1).

### 3.2. Characteristics of Included Studies

The included studies were published between 2010 and 2025 and comprised both human observational research and experimental evidence derived from animal and cellular models (Table S2). Overall, the evidence base comprised 19 studies: five human studies and fourteen experimental investigations. Within the experimental group, 12 studies were animal in vivo models, while two studies provided detailed mechanistic support using primary neuronal culture systems (rat neurons). These primary culture models were deemed essential for interrogating the direct disruption of the BDNF–TrkB signaling cascade under controlled metal exposure. Human studies covered heterogeneous populations including children, pregnant women and infants, and adult community cohorts, supporting the relevance of metal-associated neurobiological effects across different life stages [13,26,27,28,29]. In contrast, experimental evidence relied mainly on rodent models, including neonatal and adult exposure scenarios, alongside one avian model assessing neurotoxicity outcomes in a non-mammalian system [30,31,32,33,34,35,36]. Additionally, in vitro studies in primary neuronal culture systems were included, enabling the mechanistic interrogation of BDNF-related resistance pathways and neurotrophic signaling disruption under metal exposure [37,38].

Across the studies included, assessed metals and mixtures encompassed lead (Pb), mercury (Hg) (ethylmercury and methylmercury forms), cadmium (Cd), arsenic (As), manganese (Mn), and combined metal stressors, including nickel/aluminum (Ni/Al) mixtures. Lead was the most consistently examined metal across both observational and experimental settings, being evaluated in children and developmental exposure contexts and in multiple animal models [13,29,30,39,40]. Mercury exposures were primarily studied through developmental or neurodevelopmental paradigms, including prenatal environmental exposure and controlled neonatal exposure models [26,32,36]. Other metals were represented by fewer studies but contributed important mechanistic breadth, such as cadmium neurotoxicity linked to disrupted hippocampal plasticity pathways [41], arsenic-related cognitive impairment in community cohorts [28,37], and manganese-associated neurodevelopmental effects mediated through BDNF in birth cohort settings and experimental mechanistic work targeting NMDA–CREB–BDNF signaling [27,31]. Moreover, mixture and combined stressor designs highlighted the complexity of real-world exposure patterns, including Ni/Al co-exposure and Pb combined with metabolic stress induced by a high-fat diet [34,39].

Exposure assessment in human studies was largely grounded in biomonitoring, with measurements including blood lead levels, cord blood biomarkers, hair and nail metrics, and drinking water concentrations, enabling the characterization of chronic and developmental exposure scenarios in real-world settings [13,26,27,28,29]. Conversely, animal studies implemented controlled dosing regimens via oral administration or drinking water exposure, supporting causality in the evaluation of neurobiological endpoints and allowing the study of time-dependent and dose–response effects [30,31,34,35]. Notably, one experimental design explored co-exposure with a dietary modifier, demonstrating that Pb-related outcomes may be exacerbated by concurrent metabolic stressors [39].

BDNF-related outcomes were evaluated using diverse biological matrices and analytical platforms. Most investigations assessed BDNF protein levels in serum/plasma or in brain tissue, commonly relying on immunoassays such as ELISA, while several studies also quantified related upstream and downstream molecular targets, including CREB, TrkB signaling, and synaptic plasticity markers [26,31,37,39]. Beyond protein endpoints, some studies incorporated BDNF gene expression measurements, enabling a more comprehensive evaluation of neurotrophic pathway regulation across transcriptional and translational levels [29,40]. Importantly, behavioral and neurofunctional outcomes were frequently reported in animal studies, supporting functional relevance for observed BDNF alterations. Such outcomes included learning and memory performance, anxiety-like behaviors, locomotor activity changes, and social interaction impairments, indicating that metal-associated BDNF modulation may translate into measurable neurobehavioral phenotypes [30,34,35,36]. Human studies, mostly based on biomonitoring data, show variable BDNF responses, which most likely reflect variations in exposure levels, population characteristics, and developmental phases. In contrast, experimental investigations demonstrate more consistent evidence of BDNF dysregulation under controlled exposure circumstances, allowing for a more precise molecular explanation. This distinction emphasizes complementary roles of observational and experimental methods in understanding metal-induced neurotrophic changes. Collectively, the included studies demonstrate that BDNF is investigated across a wide spectrum of exposure contexts, methodological frameworks, and endpoint domains, supporting its role as a sensitive and biologically plausible marker of neurotoxicity in environmental metal exposure research.

### 3.3. Effects of Individual Metals on BDNF

Lead (Pb) was the most frequently investigated metal in the included evidence base, particularly in experimental systems, where multiple studies consistently reported BDNF dysregulation following exposure. In vivo evidence indicated that chronic or developmental Pb exposure is commonly associated with reduced hippocampal and cortical BDNF levels, frequently accompanied by neurofunctional impairment. For example, Pb exposure induced reductions in BDNF and synaptic dysfunction in developmental models, supporting a mechanistic link between early-life Pb toxicity and long-term neuroplasticity disruption [30,40]. Similarly, Pb exposure combined with metabolic stress worsened cognitive outcomes and downregulated p-CREB/BDNF signaling, highlighting BDNF as a central hub of Pb-related neurotoxicity and synaptic impairment [39]. In intervention-based models, Pb-driven cognitive deficits and BDNF downregulation were improved by neuroprotective strategies, further supporting oxidative stress and inflammatory pathways as key mediators of Pb effects [42]. Mechanistic evidence also suggested that Pb-related BDNF reductions may be partially reversible through epigenetic modulation and microglial neuroinflammation control, as sodium butyrate restored hippocampal BDNF and improved behavioral performance following Pb exposure [35]. Experimental Pb investigations consistently show decreased BDNF levels and impaired synaptic function, like cadmium and manganese. This suggests that oxidative stress and neuroinflammatory pathways are a common mechanism across various metals [43].

Human evidence for Pb showed heterogeneity, likely reflecting differences in population characteristics, developmental stage, exposure magnitude, and neurobiological compensatory capacity. In a pediatric biomonitoring setting, children with elevated blood lead reference values presented lower circulating BDNF alongside immune dysregulation, suggesting that Pb-associated neurotrophic disruption may interact with systemic inflammation pathways [13]. In contrast, another study in school-aged children reported patterns consistent with increased BDNF expression in relation to blood lead levels, which was interpreted as a possible compensatory neurotrophic response linked to neurobehavioral symptom profiles [29]. Collectively, the included Pb studies indicate that Pb exposure is frequently associated with altered BDNF signaling; however, the direction and magnitude of BDNF change may vary, potentially reflecting competing processes of neurotoxicity versus adaptive compensation in different pediatric contexts [13,29].

Evidence for mercury (Hg) encompassed both human and experimental studies, with findings collectively supporting BDNF dysregulation across exposure settings and developmental windows. In human observational research, prenatal methylmercury exposure was associated with altered cord serum BDNF patterns, providing early-life evidence that mercury may interfere with neurotrophic signaling during neurodevelopmentally sensitive periods [26]. Experimental models further demonstrated that mercury exposure can perturb BDNF-related biology through several pathways, including neuroimmune activation, neurodevelopmental disruption, and neuronal plasticity signaling. Notably, neonatal ethylmercury exposure induced structural brain changes and behavioral alterations, coinciding with microglial activation and increased cortical BDNF, suggesting that inflammatory activation and BDNF release may be mechanistically intertwined during mercury-related neurodevelopmental toxicity [36]. In contrast to the more consistently suppressive effects observed for cadmium and manganese, mercury exposure appears to induce context-dependent BDNF responses, including both upregulations associated with neuroimmune activation and the disruption of neurotrophic signaling, highlighting a more complex regulatory pattern [44].

In addition, mechanistic and systems-level designs supported multi-compartment BDNF dysregulation. For example, acute exposure comparisons between methylmercury and inorganic mercury highlighted distinct gut–brain axis profiles alongside BDNF changes across intestinal, serum, and brain-related matrices, providing evidence that mercury neurotoxicity may involve complex microbiome–metabolome–metallome interactions [32]. Cellular evidence further suggested that hippocampal neurons may exhibit BDNF-dependent resistance mechanisms against methylmercury toxicity, as experimental knockdown and neurotrophin rescue designs supported a protective role of BDNF–TrkB signaling and downstream kinase pathways [38]. Finally, one avian model indicated that selenium co-treatment alleviated mercury-induced brain damage through the activation of the BDNF/TrkB/PI3K/AKT pathway and the inhibition of inflammatory signaling, reinforcing the role of neurotrophic pathway rescue as a mechanistic endpoint in Hg neurotoxicity [33].

Cadmium (Cd) exposure was consistently associated with impaired neurotrophic signaling in experimental evidence, with several studies demonstrating reduced hippocampal BDNF levels and behavioral impairment following Cd exposure. In vivo, findings showed that cadmium-induced neurotoxicity was accompanied by oxidative stress and the downregulation of hippocampal plasticity-related proteins, including BDNF, and that neuroprotective interventions such as curcumin restored these deficits in a dose-dependent manner [41]. Similarly, cadmium exposure was linked to learning and memory deficits and broader behavioral dysfunction, with improvement following antioxidant-based mitigation strategies, supporting the hypothesis that oxidative and inflammatory signaling cascades contribute to Cd-related BDNF disruption [45].

In vitro mechanistic evidence further supported the direct disruption of neurotrophic signaling. In primary hippocampal neuronal cultures, cadmium exposure reduced BDNF–TrkB and Erk1/2 signaling and downregulated synaptic markers, while zinc supplementation exerted protective effects, indicating that the metal-induced dysregulation of zinc homeostasis may contribute to Cd-related neurotrophic impairment [37]. Collectively, the cadmium evidence consistently pointed to BDNF pathway downregulation as a core biological outcome, with oxidative stress, inflammatory signaling, and disrupted synaptic pathways likely mediating the observed effects [37,41]. Cadmium has one of the most consistent patterns of BDNF downregulation across experimental models, supporting its significant connection to oxidative stress-driven neurotoxicity and synaptic dysfunction.

Human observational evidence supported a relationship between chronic arsenic (As) exposure and reduced BDNF-related outcomes, with relevance for cognitive vulnerability in exposed communities. Specifically, higher arsenic exposure across multiple biomarkers was associated with significantly lower circulating BDNF and poorer cognitive performance, with evidence of dose-dependent relationships between exposure metrics and both neurotrophic and cognitive endpoints [28]. These findings strengthen the interpretation of BDNF as a plausible biomarker of effect for arsenic-associated neurotoxicity in environmental exposure contexts, particularly when evaluated in parallel with neurocognitive outcomes [28].

Manganese (Mn) exposure was examined in both human and experimental studies, providing convergent evidence across developmental epidemiology and mechanistic neurobiology. In a birth cohort setting, higher cord serum manganese was associated with poorer neurodevelopmental outcomes, while manganese was negatively correlated with cord serum BDNF; importantly, BDNF was positively correlated with specific neurodevelopmental dimensions, supporting a biologically plausible link between Mn exposure, BDNF modulation, and early-life neurodevelopmental function [27]. Experimental evidence supported this mechanistic plausibility, as postnatal manganese exposure downregulated CREB and reduced BDNF mRNA and protein in the hippocampus, alongside the disruption of NMDA receptor signaling components that are central to synaptic plasticity and memory-related processes [31]. Together, the Mn findings suggest that manganese neurotoxicity may involve the disruption of synaptic signaling networks upstream of BDNF regulation and that BDNF may partly mediate or modify manganese-associated developmental neurotoxicity outcomes [27,31]. Manganese, like other neurotoxic metals, such as cadmium and lead, has been associated with alterations in BDNF signaling, particularly in developmental contexts, suggesting a potentially shared molecular mechanism involving the disruption of synaptic plasticity pathways [8,46].

A limited number of studies evaluated mixture exposures or combined stressor designs, but available evidence suggested that multi-exposure contexts may exacerbate neurobehavioral impairment and BDNF-related disruption. In a mixture exposure model, combined nickel and aluminum exposure produced memory impairment and oxidative stress responses alongside reduced BDNF in brain regions relevant to learning and cognition, supporting the role of oxido-inflammatory pathways and neurotrophic depletion as a combined mechanism [34]. In a combined stressor design, Pb exposure together with a high-fat diet resulted in more severe cognitive decline and stronger reductions in CREB–BDNF signaling than either stressor alone, highlighting how metabolic context may intensify neurotoxic impacts on neurotrophic systems [39]. Overall, these findings underline the importance of moving beyond single-metal paradigms toward more realistic exposure frameworks consistent with exposome-oriented approaches [34,39].

Experimental evidence consistently indicates a decrease in BDNF levels after exposure to numerous metals, including lead, cadmium, and manganese, which is commonly associated with oxidative stress, neuroinflammation, and impaired synaptic transmission. In contrast, human studies indicate higher variation, with both decreased and, in some cases, increased BDNF levels recorded. This fact demonstrates the presence of compensatory neurotrophic responses depending on exposure timing, dose, and demographic characteristics. Mercury-related findings demonstrate context-dependent effects, with evidence of both BDNF upregulation associated with neuroimmune activation and the disruption of neurotrophic pathways throughout developmental windows. Despite these variations, BDNF appears to function as a key integrative node connecting environmental exposure to brain dysfunction in a shared molecular framework that develops across metals. These data underscore the need to consider both exposure context and study design when interpreting BDNF as a biomarker of metal-induced neurotoxicity [5,47].

### 3.4. Study Quality and Risk of Bias Assessment

Quality appraisal was performed separately for human and experimental evidence. Human observational studies were evaluated using the Newcastle–Ottawa Scale (NOS) and categorized as good, fair, or poor quality, while experimental animal and supportive in vitro studies were assessed using SciRAP criteria and categorized as high, medium, or low reliability.

Among the five human studies, three were rated as good quality and two as fair quality, with no studies classified as poor. Higher-quality evidence was mainly derived from birth cohort and population-based designs characterized by robust exposure assessment through biomonitoring matrices and more comprehensive handling of potential confounders [26,27,28]. In contrast, the two fair-quality studies were limited primarily by cross-sectional design, modest sample size, and less detailed reporting of participant selection procedures and confounder adjustment strategies [13,29]. Despite these limitations, all human studies employed biologically relevant exposure metrics (e.g., blood, cord serum, water, hair, or nails) and BDNF outcome assessment approaches, supporting the overall interpretability of the observational evidence base [13,26,28].

Across the experimental evidence base, most animal studies were rated as medium-to-high reliability, reflecting clear exposure characterization, validated outcome assessment strategies, and consistent reporting of key mechanistic endpoints relevant to BDNF dysregulation. Strong mechanistic designs with integrated behavioral outcomes were observed in several studies, strengthening the biological plausibility and functional interpretation of BDNF modulation following exposure [34,35,36,39]. However, methodological reporting gaps were frequently noted in domains including randomization procedures, allocation concealment, and the blinding of outcome assessment, which may increase the risk of performance and detection bias across experimental findings [30,41,45]. Notably, several studies achieved high reliability due to strong exposure quantification and comprehensive multi-endpoint outcome assessment, including advanced mechanistic profiling (e.g., epigenetic regulation and neuroinflammatory pathways) and/or combined exposure paradigms [32,35,39,40].

Supportive in vitro studies demonstrated high mechanistic reliability, as they directly interrogated BDNF-related signaling pathways under tightly controlled exposure conditions and enabled causal inference regarding neurotrophic pathway disruption or neuroprotective signaling. For instance, mechanistic cell culture systems provided evidence for the direct cadmium-associated impairment of BDNF-TrkB signaling and synaptic pathway components, as well as mercury-related BDNF-dependent resistance mechanisms in hippocampal neurons [37,38]. Nevertheless, although cellular models offer strong mechanistic certainty, their external validity in relation to organism-level behavioral outcomes is inherently limited relative to in vivo experimental designs.

Overall, quality appraisal supported a biologically plausible association between metal exposure and altered BDNF regulation across both observational and experimental evidence, while emphasizing methodological variability and incomplete reporting of bias-mitigating practices as key considerations when interpreting the strength and consistency of the evidence [26,28,35,36] (Tables S3 and S4).

## 4. Discussion

This systematic review synthesized evidence from 19 eligible human and experimental studies examining the associations between environmental metal exposure and alterations in brain-derived neurotrophic factors (BDNFs). Overall, the evidence supports a biologically plausible relationship between toxic metal exposure and the disruption of neurotrophic signaling, although the direction and magnitude of BDNF changes were not fully uniform across metals, biological matrices, and exposure windows. Importantly, BDNF alterations were rarely isolated outcomes; instead, they were frequently reported alongside oxidative stress, neuroinflammation, synaptic dysfunction, and in multiple animal studies, measurable impairments in learning and memory, anxiety-like behavior, or social interaction [34,35,36,39]. Taken together, these converging observations support BDNF as a mechanistically relevant biomarker candidate for neurotoxicity and impaired neuroplasticity in metal exposure contexts [5].

BDNF plays a central role in neuronal survival and synaptic plasticity through TrkB signaling, and reduced BDNF availability has been implicated in impaired neurogenesis, maladaptive plasticity, and mood-related vulnerability. Within the included evidence base, a recurring mechanistic pattern was observed across metals: exposures that promoted mitochondrial dysfunction, reactive oxygen species (ROS) generation, and inflammatory activation were also frequently linked to BDNF downregulation or the broader disruption of neurotrophic pathways. This convergence is consistent with a framework in which oxidative and inflammatory stress acts as an upstream biological pressure capable of suppressing neurotrophic signaling and contributing to downstream cognitive and behavioral impairment [30,34,41].

As the most frequently investigated metal, lead (Pb) consistently demonstrates a capacity to reduce hippocampal and cortical BDNF in experimental models of chronic or developmental exposure, typically alongside synaptic protein alterations and oxidative stress [30,40]. These neurotrophic deficits are often exacerbated by real-world co-stressors; for instance, Pb combined with a high-fat diet produces more pronounced cognitive impairment and CREB–BDNF suppression than single-stressor models [39]. The mechanistic link is further supported by intervention studies where neuroprotective agents or epigenetic modifiers, such as sodium butyrate, restored behavioral performance by modulating BDNF through ACSS2/H3K9ac-linked pathways and reduced neuroinflammation [35,42]. However, human pediatric evidence remains heterogeneous and underscores a critical interpretive challenge: while some biomonitoring data link elevated Pb to lower circulation BDNF and immune dysregulation [13], other studies observe increased BDNF expression alongside neurobehavioral symptoms [29]. This suggests that BDNF levels reflect a complex interface between neurotoxic suppression and adaptive compensatory signaling, which varies significantly based on developmental timing, exposure intensity, and the specific biological matrix being measured [5].

Evidence for mercury (Hg) highlights a strong emphasis on early-life vulnerability, with prenatal methylmercury exposure linked to altered cord serum BDNF during critical neurodevelopmental windows [26]. Mechanistically, neonatal ethylmercury exposure has been associated with microglial activation and increased cortical BDNF alongside behavioral impairments, suggesting that immune-mediated BDNF release constitutes a core component of mercury-related neurodevelopmental disruption [36]. Further resolution provided by multi-omics data reveals distinct neurotoxicity signatures for methylmercury versus inorganic mercury, reflected in gut–brain axis profiles and BDNF fluctuations across biological matrices [32]. Notably, BDNF–TrkB signaling may also facilitate hippocampal resistance against methylmercury toxicity, positioning BDNF not only as a sensitive marker of perturbation but also as an endogenous mediator of compensatory or protective neurotrophic capacity [38].

Cadmium (Cd) evidence was largely derived from experimental models and showed relatively consistent directionality, supporting the suppression of neurotrophic signaling within hippocampal pathways. Cd exposure was repeatedly linked to oxidative stress and behavioral impairment with corresponding reductions in hippocampal BDNF, while antioxidant and neuroprotective interventions partially restored BDNF-associated outcomes [41,45]. Mechanistic in vitro evidence further suggested that Cd can directly disrupt BDNF–TrkB and ERK-related signaling and downregulate synaptic markers, with zinc supplementation providing partial rescue, indicating that metal dyshomeostasis and disrupted synaptic zinc signaling may contribute to neurotrophic vulnerability [37]. This convergence supports a model in which Cd toxicity intersects oxidative pathways and plasticity signaling, ultimately leading to impaired BDNF-mediated neuroadaptation.

Although fewer studies examined arsenic and manganese, available human evidence provides important relevance for environmentally exposed populations. Chronic arsenic exposure, assessed across multiple exposure biomarkers, was associated with lower circulating BDNF and poorer cognitive performance, supporting the potential utility of BDNF as an effect marker reflecting neurobehavioral vulnerability in real-world settings [28]. Likewise, prenatal manganese exposure was associated with infant neurodevelopment outcomes, with cord serum Mn negatively correlated with cord BDNF and BDNF positively related to developmental scores, indicating that neurotrophic signaling may mediate or modify early-life vulnerability to Mn exposure [27]. The experimental findings supported mechanistic plausibility, linking postnatal Mn exposure to the disruption of NMDA–CREB pathways and reduced hippocampal BDNF expression [31].

A limited number of studies examined mixture exposures or combined stressors, but findings were notable for highlighting exposure realism and potential synergy. Ni/Al co-exposure induced stronger memory impairment and more pronounced BDNF reductions than single-metal exposure, consistent with shared oxido-inflammatory pathways converging on neurotrophic regulation [34]. Likewise, Pb combined with a high-fat diet produced additive effects on cognitive impairment and neurotrophic disruption, emphasizing that BDNF may be sensitive to multi-domain exposure patterns that reflect real-world exposome contexts [39]. These findings strengthen the argument for integrated approaches that consider chemical mixtures and lifestyle or metabolic modifiers rather than evaluating metals in isolation.

An important interpretive challenge across studies concerns differences in biological matrices and analytical platforms used for BDNF assessment. Circulating BDNF is influenced by platelet dynamics, systemic inflammatory and metabolic states, and short-term physiological modulation, whereas region-specific brain BDNF measurements provide a closer mechanistic linkage to synaptic plasticity and neurobehavioral function [5]. However, the use of peripheral BDNF as a surrogate for central neurotrophic status is supported by evidence that BDNF can cross the blood–brain barrier in both directions via high-capacity transport systems. Crucially, comparative studies across multiple species have demonstrated that blood BDNF concentrations significantly correlate with brain tissue levels, validating the use of circulating protein as a representative proxy for central nervous system health [48]. Therefore, discordant findings between peripheral and central BDNF do not necessarily represent contradiction; rather, they may reflect differences in what each matrix captures biologically, as well as differences in exposure windows and developmental timing. This supports the value of integrating BDNF with complementary endpoints (e.g., oxidative stress, inflammatory mediators, synaptic proteins, and omics-derived pathway signatures) to improve interpretability and strengthen causal inference [32,35].

**Figure 2:**
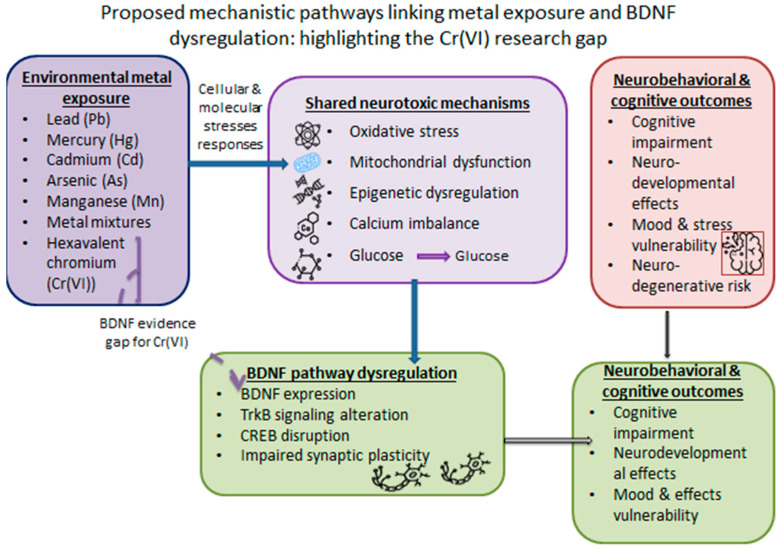
A proposed mechanistic model relates general metal exposure to dysregulation of neurotrophic signaling pathways. Multiple metals, including Pb, Hg, Cd, As, Mn, and metal mixtures, share processes such as oxidative stress, mitochondrial dysfunction, neuroinflammation, and epigenetic dysregulation, each of which may affect BDNF-TrkB signaling and synaptic plasticity. The resulting neurotrophic disturbance may influence cognitive, neurodevelopmental, and neurobehavioral consequences. Despite significant molecular plausibility, no studies have explicitly investigated BDNF-related consequences following hexavalent chromium [Cr(VI)] exposure, highlighting a significant research gap.

A key finding of this review is the complete absence of eligible studies evaluating BDNF outcomes in relation to hexavalent chromium [Cr(VI)] exposure. This gap is particularly striking given the established evidence that Cr(VI) induces the same upstream biological processes, including oxidative stress, mitochondrial dysfunction, inflammatory activation, DNA damage, and metabolic disruption, that are repeatedly implicated in BDNF dysregulation across other toxic metals [10,49,50]. Furthermore, Cr(VI) has been shown to selectively accumulate in the hippocampus and induce age- and sex-dependent metal dyshomeostasis, mechanisms that plausibly overlap with the regulation of neurotrophic signaling and synaptic plasticity. Recent mechanistic work also indicates that Cr(VI) perturbs metabolic pathways in astrocytes, reinforcing the biological plausibility for downstream neurotrophic disruption [51]. It is biologically plausible that Cr(VI) could affect neurotrophic signaling by routes like those found for Pb, Cd, Hg, and Mn, given this mechanistic convergence (Figure 2).

The lack of evidence for Cr(VI)-BDNF impacts not only biomarker evaluation but also the integration of chromium neurotoxicity into mechanistic frameworks such as Adverse Outcome Pathways (AOPs) and exposome-oriented risk assessment models. This gap is particularly important considering growing environmental and occupational exposure concerns and the expanding use of multi-omics approaches capable of capturing early neurobiological perturbations. Addressing this gap is critical to deciding if BDNF is a sensitive biomarker of impact in chromium-exposed populations and enabling the development of mechanism-based neurotoxicity models that connect environmental stresses to neurodevelopmental and neurobehavioral outcomes.

The classification of BDNF as a promising biomarker, despite the noted variability across matrices and the limitations of cross-sectional data, is grounded in its role as a sensitive indicator of sub-clinical neurobiological stress. Unlike conventional biomarkers of exposure, BDNF serves as a biomarker of effect, reflecting the functional state of neuronal plasticity before irreversible structural damage occurs. Its promise is further supported by its mechanistic centrality; evidence indicates that BDNF acts as a convergence point for multiple metal-induced insults, including reactive oxygen species (ROS) generation, mitochondrial dysfunction, and neuroinflammatory signaling [52]. Therefore, the utility of BDNF lies in its integration within multi-level diagnostic frameworks, such as Adverse Outcome Pathways (AOPs), where it links molecular environmental stressors to downstream cognitive outcomes.

Future research should aim to clarify whether BDNF can serve as a robust biomarker of effect in metal-associated neurotoxicity by improving comparability across designs and increasing mechanistic resolution. Priority directions include: (i) harmonized exposure characterization using validated biomarkers (blood/urine/hair/nails), including metal speciation when relevant; (ii) standardized BDNF assessment (serum vs. plasma vs. brain tissue) with explicit reporting of pre-analytical conditions and assay platforms; (iii) the integration of BDNF with complementary endpoints including oxidative stress markers, inflammatory mediators, mitochondrial function, synaptic proteins, and omics signatures [5,35]. Human studies should further incorporate longitudinal sampling and validated neurodevelopmental or neuropsychiatric outcomes with rigorous confounder control. Experimental work should adopt environmentally relevant exposure windows, include brain region specificity, and integrate behavioral testing with molecular endpoints. Importantly, Cr(VI)-focused studies should explicitly include BDNF and related TrkB/CREB signaling targets, supported by omics-based approaches, to accelerate mechanism-based frameworks and biomarker development [8].

To evaluate the internal validity of the included evidence, study-specific quality assessment tools were utilized: the Newcastle–Ottawa Scale (NOS) for human observational studies and the SciRAP tool for experimental models. The NOS was selected for its validated focus on selection and comparability in clinical research [24], while SciRAP was chosen specifically for its ability to rigorously evaluate the technical reliability and reporting quality inherent to toxicological and risk assessment studies [25]. By employing these distinct frameworks, we ensured a standardized yet specialized appraisal that accounts for the unique methodological requirements of both epidemiological and laboratory-based neurotoxicity research.

Several limitations warrant consideration. First, substantial heterogeneity in study design, including varying exposure metrics, developmental windows, and experimental dosages, limited cross-study comparability and precluded quantitative meta-analysis. Second, differences in BDNF measurement methods and biological matrices represent a significant source of variability; circulating BDNF is highly sensitive to pre-analytical handling and the choice between serum and plasma, which may mask subtle neurotoxic effects depending on platelet degranulation. Third, many human studies were cross-sectional, restricting causal inference, and frequently lacked rigorous control for potential confounding factors. Peripheral BDNF is a dynamic biomarker influenced by lifestyle variables such as body mass index (BMI), physical activity, and smoking status, which were not consistently adjusted for across the evidence base. Fourth, experimental designs varied in dose and duration, which may limit generalizability to chronic, low-level environmental settings. Finally, the absence of Cr(VI)-specific evidence prevents direct conclusions regarding chromium’s neurotrophic effects and remains an unaddressed research gap.

## 5. Conclusions

This systematic review synthesizes evidence from human observational studies and experimental models indicating that exposure to multiple environmental metals, most consistently lead (Pb), mercury (Hg), cadmium (Cd), arsenic (As), and manganese (Mn), is associated with BDNF dysregulation and the disruption of neurotrophic signaling. Across the included evidence base, BDNF alterations frequently co-occurred with biological signatures of oxidative stress, mitochondrial dysfunction, neuroinflammation, synaptic impairment, and epigenetic modulation, supporting mechanistic pathways through which metal exposure may compromise neuroplasticity and contribute to adverse neurobehavioral outcomes. Despite overall mechanistic convergence, the direction and magnitude of BDNF changes were not fully uniform across studies, reflecting heterogeneity in exposure characterization, biological matrices (peripheral versus brain BDNF), life-stage vulnerability, and study design. Nevertheless, the collective findings reinforce BDNF as a biologically relevant and potentially informative biomarker of effect for environmental metal-related neurotoxicity, particularly when interpreted alongside complementary molecular and functional endpoints. Notably, no eligible studies evaluated BDNF outcomes in relation to hexavalent chromium [Cr(VI)], representing a significant research gap given that Cr(VI) shares many upstream neurotoxic mechanisms with the metals reviewed here. Future research should prioritize addressing this void by integrating standardized neurotrophic biomarkers with exposome-oriented designs and Adverse Outcome Pathway (AOP) frameworks to improve evidence-based risk assessment for chromium and other neurotoxic metal exposures.

## Data Availability

No new data were created or analyzed in this study.

## Supplementary Materials

The following supporting information can be downloaded at: https://www.mdpi.com/article/10.3390/jox16020059/s1, Table S1: Search terms used for systematic review on databases; Table S2: Main characteristics of research evaluating environmental metal exposure and BDNF-related outcomes included in the systematic review; Table S3: Quality assessment of included human observational studies using the Newcastle–Ottawa Scale (NOS); Table S4: Quality and reliability assessment of experimental animal and in vitro studies using SciRAP criteria.
