# Supplementary material for: Environmental Metal Exposure and Brain-Derived Neurotrophic Factor (BDNF): A Systematic Review of Human and Experimental Evidence
Source: J Xenobiot. 2026 Apr 2;16(2):59. doi: 10.3390/jox16020059 (PMC13117395; doi:10.3390/jox16020059)
Supplement: Supplementary file 1 [file jox-16-00059-s001.zip › jox-4197178-supplementary.pdf]

Environmental Metal Exposure and Brain-Derived Neurotrophic Factor (BDNF): A Systematic Review of Human and Experimental Evidence

Maria-Nefeli Georgaki, Despoina Ioannou, Elpis Chochliourou, Kanellos Skourtsidis, Theodora Papamitsou and Dimosthenis Sarigiannis

Table S1. Search terms used for systematic review on databases		
Category	Sub-domain	Search terms
BDNF - Neurotrophic - Mental Health	Core BDNF terms	BDNF; brain-derived neurotrophic factor; brain derived neurotrophic factor; neurotrophic factor; neurotrophin
	Signaling pathways	neurotrophin signaling; TrkB; NTRK2; CREB; BDNF signaling
	Molecular expression	BDNF mRNA; BDNF protein; serum BDNF; plasma BDNF
	Epigenetics & genetics	BDNF promoter methylation; BDNF epigenetics; Val66Met polymorphism
	Neurobiology	synaptic plasticity; neuronal plasticity; neurogenesis; hippocampus; neurodevelopment
	Cognitive outcomes	cognitive function; cognitive impairment; memory deficits
	Mental health outcomes	mood disorders; depression; depressive-like behavior; anxiety-like behavior; neuropsychiatric outcomes; stress response
	Neurotoxicity/behavior	neurobehavioral toxicity
Metal Terms	General metal exposure	metal exposure; metal toxicity; heavy metals; trace metals; essential metals; neurotoxic metals
	Individual metals	arsenic; cadmium; lead; mercury; manganese; copper; zinc
	Chromium-specific	chromium; total chromium; trivalent chromium; chromium VI; hexavalent chromium; Cr(VI); Cr6+; chromium contamination
	Environmental context	metal mixtures; drinking water contamination; groundwater contamination; environmental metals
Exposure - Toxicology Terms	Oxidative mechanisms	oxidative stress; redox imbalance; reactive oxygen species; ROS
	Mitochondrial & cellular effects	mitochondrial dysfunction; ATP depletion; apoptosis
	Genetic & epigenetic damage	DNA damage; DNA methylation; epigenetic modification
	Inflammation	inflammatory signaling; cytokines; NF-κB; IL-6; TNF-α; neuroinflammation; microglial activation
	Neurotransmission	excitotoxicity; calcium dysregulation; glutamatergic signaling; GABAergic signaling
	Functional outcomes	synaptic dysfunction; neurotoxicity; cellular stress response
	Biomarkers	biomarker

Table S2. Main characteristics of research evaluating environmental metal exposure and BDNF-related outcomes included in the systematic review

ID	Study ID	Country	Design	Population - Species	Sample Size	Metals	Exposure Type	Exposure Measure	BDNF Measure	Method	Main Findings	Effect Size	Mechanistic Notes	Behavioral Outcomes	Comments
1	[13]	China	Human observational (case-control / cross-sectional by BLRV threshold)	Children (1–5 years)	n=100 (50 elevated BLRV ≥3.5 μg/dL, 50 low BLRV <3.5 μg/dL)	Lead (Pb)	Environmental (blood lead)	Blood lead reference value (BLRV), flame atomic absorption spectroscopy	Plasma BDNF concentration	Cytokine antibody array (174 cytokines) + validation by ELISA	Elevated BLRV group showed decreased BDNF + multiple cytokine changes (IL-6/IL-8/IL-17 ↑; IL-7/IL-10 ↓ etc.)	Not extracted as exact numeric	Inflammation + neurotrophin signaling involvement (GO/KEGG enrichment includes neurotrophin pathway); immune dysregulation in Pb toxicity	Not direct neurobehavioral tests	Strong "biomarker discovery/validation" structure
2	[25]	South Korea	Experimental in vivo (neonatal mouse model) + in vitro cell models (microglia/neurons/astrocytes)	Neonatal mice	Not clearly extractable	Mercury (ethylmercury, EtHg)	Experimental exposure	EtHg dose exposure (designed to mimic potential human exposure levels)	BDNF expression/release (cortex)	Neurochemical + morphological/brain development assessments + microglial activation + BDNF-related assays	EtHg exposure caused brain structural changes (brain size/cortical thickness ↑) + social interaction impairments and was linked with microglial activation and elevated BDNF in cortex	Not extracted	Microglial activation → MMP activation → BDNF release → neurodevelopmental disruption	Social interaction / behavioral pattern impairments	Very good mechanistic study on Hg–BDNF axis, especially early-life exposure.

3	[26]	Faroe Islands cohort (Nordic setting)	Human observational cohort (birth cohort 1999–2001)	Newborns (umbilical cord serum)	n=395 singleton births (206 boys, 189 girls)	Mercury (methylmercury exposure via diet) (+ assessed PCB too)	Prenatal environmental exposure (maternal diet/contaminants)	Hg in cord blood (atomic absorption), maternal hair mercury (long-term)	Serum BDNF in cord blood	Two-site sandwich ELISA	MeHg exposure associated with decreased BDNF (dose-dependent) in girls born to nonsmoking mothers. Maternal smoking increased BDNF in girls but also enhanced MeHg-related decrease.	Reported as concentration-dependent decrease	Developmental neurotoxicity; reduced BDNF may impair compensatory neurodevelopmental responses	Not directly measured (suggested predictive marker relevance)	strong human prenatal exposure study
4	[42]	India	Experimental in vivo (rat model)	Adult male Wistar rats	n=30 total (5 groups × 6 rats/group)	Lead (Pb; lead acetate)	Experimental oral exposure	Lead acetate 100 mg/kg/day × 28 days + serum lead levels	Hippocampal BDNF	Behavioral test (Novel Object Recognition) + biochemical assays (BDNF, Nrf2, IL-6, GFAP, neurotransmitter	Lead caused cognitive impairment + ↓ acetylcholine/glutamate + ↓ BDNF + ↑ IL-6/GFAP + neuronal damage. NAC and naringin restored BDNF and cognition (dose-	Not extracted as numeric (clear group differences)	Oxidative stress (Nrf2), neuroinflammation (IL-6, GFAP), neurotrophic support (BDNF)	Recognition memory deficits (NOR test) improved by NAC/naringin	intervention/therapy study;mechanistic support Pb→BDNF→cognition.

										s) + histology	dependent for naringin).				
5	[35]	China	Experimental in vivo (mouse) + human observational component (butyric acid vs lead exposure & cognition mediation)	Male C57BL/6J mice (4–5 weeks) + human participants (not fully described in snippet)	mice n=8/group (multiple groups); humans N not visible in snippet	Lead (Pb, lead chloride)	Experimental chronic exposure in drinking water + human exposure grouping	$PbCl_2$ 50/100/200 mg/L × 12 weeks; Pb measured by ICP-MS in blood	Hippocampal BDNF expression (downstream of histone acetylation)	Morris water maze + open field test + cytokine ELISAs + SCFAs measurement (GC-MS) + mechanistic assays (ACSS2 / H3K9ac / STAT3 microglia)	Pb caused learning/memory deficits + anxiety-like behavior. Pb lowered serum butyrate. Sodium butyrate improved behavior and restored BDNF via ACSS2/H3K9ac and reduced microglial neuroinflammation (STAT3). Humans: butyrate was lower in high Pb exposure and	Mediation contribution 27.57% reported (numeric); behavioral improvements significant (plots).	Epigenetic regulation (histone acetylation) + neuroinflammation + neuronal acetyl-CoA metabolism → BDNF recovery	Morris water maze + open field (memory/anxiety) improved with butyrate	Very strong mechanistic study that “ties” Pb exposure to gut metabolites/epigenetics/BDNF

											mediated Pb–cognition association (~27.57%).				
6	[41]	India	Experimental in vivo (mouse)	Swiss Albino mice		Cadmium (Cd)	Experimental	Cd 2.5 mg/kg (pre-treated model)	Hippocampal BDNF (protein)	Behavioral + biochemical oxidative stress + hippocampal proteins (BDNF, CREB, DCX, Synapsin II) + histology	Cd caused behavioral impairment + oxidative stress + ↓ hippocampal neurogenesis proteins (incl. BDNF). Curcumin restored deficits dose-dependently (best 160 mg/kg).	Not extracted numeric (significant dose-response)	CREB–BDNF signaling + neurogenesis restoration; anti-oxidant/anti-inflammatory	Behavioral impairment reversed (exact tests not listed in snippet)	Strong mechanistic "Cd → ↓BDNF/CREB → neurogenesis↓" + nutraceutical rescue.

7	[34]	Nigeria (+ collaboration Serbia)	Experimental in vivo (rat)	Male Sprague Dawley rats	n=28 rats (4 groups)	Nickel (Ni) + Aluminium (Al) (binary mixture)	Experimental oral mixture exposure	Al 1 mg/kg, Ni 0.2 mg/kg, mixture 90 days (3×/week)	BDNF levels in hippocampus + frontal cortex	Barnes maze + brain tissue metal levels (AAS) + oxidative stress markers (MDA/CAT/SOD/GPx/GSH) + COX-2/AChE + BDNF/NGF (ELISA)	Ni, Al, and especially mixture → memory impairment + oxidative stress + COX-2 ↑ + BDNF ↓ (region-specific) + AChE disruption	Not extracted numeric	Oxido-inflammatory pathway + COX-2/AChE/BDNF signaling axis	Barnes maze memory impairment	Strong mixture exposure study (exposome-relevant)
8	[45]	Pakistan	Experimental in vivo (rat)	Male Albino-Wistar rats	n=32 rats (8/group)	Cadmium (Cd; CdCl2)	Experimental (subacute) + nutraceutical protection	CdCl2 5 mg/kg (last 3 days), thymol 40 mg/kg orally for 14 days	Hippocampal BDNF levels	Behavioral battery + hippocampal BDNF measurement	Cd induced learning/memory deficits + anxiety/motor incoordination/depression-like signs; thymol improved behavior and	Not extracted numeric	Neuroprotection via plasticity/BDNF enhancement (antioxidant rationale)	Learning & memory + anxiety + motor + depression endpoints improved	Clear behavioral relevance + BDNF hippocampus endpoint

											upregulated hippocampal BDNF				
9	[33]	China	Experimental in vivo (chicken)	Chickens	Not clearly extractable	Mercury (HgCl2) + Selenium (Na2SeO3)	Experimental exposure + protective co-treatment	HgCl2-induced neurotoxicity model (dose not in snippet)	BDNF/TrkB/PI3K/AKT pathway activation	Histopathology + oxidative stress (MDA, T-SOD, GSH-Px, T-AOC) + apoptosis/inflammation markers + pathway analysis	HgCl2 caused brain damage + oxidative stress + apoptosis/inflammation; selenium alleviated injury by activating BDNF/TrkB/PI3K/AKT and inhibiting NF-κB	Not extracted numeric	Antioxidant/anti-inflammatory protection + BDNF signaling rescue	Not reported (tissue/mechanistic study)	Strong signaling/mechanistic paper; no behavioral tests
10	[32]	China	Experimental in vivo (rat) – acute exposure + multi-omics	Male Sprague Dawley rats	Not clearly extractable	Mercury (MeHg vs inorganic Hg)	Experimental acute oral exposure	MeHg vs IHg (24h) + Hg distribution intestine + multi-omics (metallomics/metabolomic	BDNF in intestine, brain, and serum	Gut microbiome + metabolomics + metallomics +	MeHg vs IHg caused distinct fecal microbiome/metabolome profiles; differences	Not extracted numeric	Gut–brain axis disruption + omics-driven neurotoxicity signatures	Not reported (screening/omics focus)	Excellent exposome/omics mechanistic study

								s/microbiome)		neurotransmitter-related metabolites	supported by tissue Hg distribution and BDNF changes in intestine/brain/serum				
11	[38]	Japan	Experimental in vitro (primary neurons; CCNs vs HiNs comparison)	Cultured fetal rat neurons (cerebrocortical neurons vs hippocampal neurons)	n=6 (per experiment; reported mean±SEM)	Mercury (methylmercury, MeHg)	Experimental exposure	MeHg chloride 0.25–10 μM (24 h exposure)	BDNF protein expression (neurons)	Primary neuron culture + cell viability (MTS) + siRNA knockdown (BDNF/NT3/NT4/5) + western blot (BDNF/Trk/phospho-kinases) + pathway analysis	BDNF specifically expressed in hippocampal neurons (HiNs) and contributes to resistance against MeHg toxicity; BDNF knockdown reduced resistance; exogenous BDNF/NT4/5 suppressed MeHg neurotoxicity	Not extracted numeric (IC50/viability differences shown)	BDNF–TrkB protective signaling; p44/42 MAPK pathway activation contributes to MeHg resistance	Not reported (cellular model)	Strong mechanistic evidence: BDNF as protective factor against MeHg neurotoxicity

12	[30]	Bangladesh	Experimental in vivo (gestational/lactational exposure)	Offspring rats (maternal exposure model)	Not clearly extractable (maternal divided into control vs Pb-exposed; offspring assessed)	Lead (Pb; lead acetate)	Developmental exposure (maternal drinking water during pregnancy & lactation)	0.1% (w/v) Pb acetate in drinking water; Pb measured in milk and offspring brain (AAS)	Brain BDNF protein level (offspring brain)	Biochemical assays (Pb, lipid peroxide, TNF-α, cytochrome c) + western blot for synaptic proteins (SNAP-25, PSD-95) + BDNF/TrkB + locomotor activity (open field tracking software)	Maternal Pb exposure increased oxidative stress/inflammation/apoptosis markers and decreased synaptogenesis/cognition-related proteins including BDNF and TrkB in offspring brains	Not extracted numeric	Oxidative stress + TNF-α increase + apoptosis (cytochrome c) with impaired BDNF–TrkB signaling	Locomotor activity deficits in pups (open field)	Strong developmental Pb model linking milk exposure → offspring brain BDNF↓ + neurodevelopment disruption

13	[31]	China	Experimental in vivo (postnatal exposure)	Newborn Sprague–Dawley rats (hippocampus)	n=10/group (5 male + 5 female)	Manganese (Mn2+)	Postnatal experimental exposure (i.p.)	MnCl2·4H2O i.p. daily for 3 weeks: 0 / 10 / 20 / 30 mg/kg	BDNF mRNA + protein in hippocampus	RT-qPCR + western blot for NMDA receptor subunits (NR1/NR2A/NR2B) + CREB + BDNF in hippocampus	Mn exposure decreased NMDA receptor signaling components and downregulated CREB and BDNF mRNA/protein in hippocampus	Not extracted numeric	Disrupted NMDA receptor signaling → CREB downregulation → BDNF reduction as mechanistic pathway	Not directly tested (molecular endpoints only)	Good mechanistic pathway paper for Mn neurotoxicity via NMDA–CREB–BDNF axis
14	[37]	Tunisia / France	Experimental in vitro (primary hippocampal neurons)	Primary rat hippocampal neuronal cultures (embryonic day 17.5)	Multiple independent experiments (triplicate; n not clearly extractable)	Cadmium (Cd) + Zinc (Zn)	Experimental exposure ± Zn supplementation	CdCl2 10 or 25 μM (24–48 h) ± ZnCl2 30 μM (protective)	BDNF–TrkB signaling (protein/gene expression)	MTS viability + apoptosis/necrosis markers (Annexin V/7-AAD) + RT-qPCR + western blot; assessed	Cd exposure reduced BDNF–TrkB and Erk1/2 signaling and downregulated synaptic markers; Zn supplementation partially/fully protected; ZnT3 involved in mechanism	Not extracted numeric	Cd disrupts Zn homeostasis; synaptic Zn transporter ZnT3 contributes; BDNF–TrkB + Erk1/2 signaling impairment	Not reported (cell culture)	Strong in vitro mechanistic evidence: Cd → synaptic dysfunction + BDNF–TrkB down; Zn partially rescues

										synaptic proteins (NR2A, PSD-95) + ZnT3 transporter involvement					
15	[28]	Bangladesh	Human observational (cross-sectional)	Adults 18–60 years (rural Bangladesh; low vs high arsenic areas)	Total n=693; serum BDNF measured in n=490	Arsenic (As)	Environmental (chronic drinking water exposure)	As in drinking water + hair + nails (ICP-MS); exposure dose-dependent across metrics	Serum BDNF (sBDNF)	Human MMSE (Bangla version) + ELISA (R&D Systems) for sBDNF; regression models with confounder adjustment	MMSE score and serum BDNF were significantly lower in high-As areas; both decreased dose-dependently with increasing As in water/hair/nails; MMSE positively correlated with sBDNF	Not extracted numeric (dose-response + adjusted regression significant)	Chronic As may reduce BDNF as part of biochemical basis of cognitive impairment; supports BDNF as biomarker for As-related neurotoxicity	Yes (cognitive impairment via MMSE)	Strong large human study with multiple exposure biomarkers (water/hair/nails)

16	[29]	India	Human observational (cross-sectional)	School-going children 9–15 years (Indian children)	N=72	Lead (Pb)	Environmental (blood lead)	Blood lead levels by GFAAS (graphite furnace AAS); median BLL 4.95 μg/dL	Serum BDNF levels + BDNF mRNA expression	CPMS (Childhood Psychopathological Measurement Schedule) + Real-Time PCR (BDNF mRNA) + ELISA (serum BDNF)	BLL positively correlated with CPMS neurobehavioral scores and with BDNF expression; BDNF mRNA and serum BDNF higher in high-BLL children (not always statistically significant)	Not extracted numeric (correlations significant for CPMS & BLL; BDNF ↑ trend)	Possible compensatory BDNF response or role in Pb-induced neurobehavioral alterations; suggests involvement of neurotrophic signaling in Pb effects	Yes (CPMS neurobehavior outcomes; depression higher in boys with high BLL)	Good human child neurobehavior + BDNF gene/protein study (direction differs from some Pb studies)
17	[39]	China	Experimental in vivo (rat)	Male Sprague Dawley rats (4-week-old)	n=40 total (4 groups × 10)	Lead (Pb) + High-fat diet (HFD)	Combined exposure (metal + dietary stressor)	0.2% lead acetate in drinking water × 12 weeks ± HFD	BDNF protein levels (hippocampus/cortex)	Morris water maze + Nissl staining + western blot (SIRT1/NMDARs/p-CREB/B	Pb and HFD each caused cognitive deficits and neuronal damage; co-exposure worsened outcomes; BDNF and p-CREB decreased (greater	Not extracted numeric (group differences significant; additive effect)	CREB–BDNF signaling hub + synaptic plasticity impairment + brain insulin signaling disruption (IRS-1/PI3K/Akt)	Yes (MWM cognitive impairment)	Excellent mixture/stressor interaction study (Pb + lifestyle factor) for "realistic exposures"

										DNF/synaptic proteins) + insulin signaling proteins (IRS-1/PI3K/Akt)	decrease with combined exposure); synaptic proteins and insulin signaling impaired				
18	[27]	China	Human observational (prospective birth cohort)	Pregnant women + infants (LW Birth Cohort); children assessed at 1 year	n=377 for Mn+BDNF (subset with sufficient serum volume)	Manganese (Mn)	Prenatal exposure (cord serum Mn)	Cord serum Mn by ICP-MS; median Mn 3.4 μg/L	BDNF in cord serum	Cord serum Mn & BDNF (ELISA) + Gesell Developmental Inventory (DQ: motor/adaptive/language/social) + regression models with confounder	Prenatal Mn associated with poorer neurodevelopment (gross motor & personal-social); Mn negatively correlated with BDNF; BDNF positively correlated with personal-social score; interaction Mn×BDNF significant	Not extracted numeric (β coefficients reported; significant interaction)	BDNF plays important role in Mn-induced cognitive impairment; suggests neurotrophic mediation/moderation mechanism	Yes (Gesell DQ neurodevelopment outcomes at 1 year)	Strong human prenatal cohort connecting exposure biomarker → BDNF → infant development

										adjustment					
19	[40]	Poland	Experimental in vivo (perinatal exposure rat model)	Wistar rat offspring (28-day old pups; exposure gestation → lactation)	Ultrastructure: n=8 (4 control, 4 Pb); Gene expression: n=16 (8/group); Protein WB: n=16 (8/group)	Lead (Pb; lead acetate PbAc)	Perinatal developmental exposure (maternal drinking water)	0.1% Pb acetate in drinking water from gestation day 1 until weaning (PND21); pups then water until PND28; Pb-B 6.86 μg/dL (Pb) vs 0.93 μg/dL (control) (GFAAS)	BDNF level in brain regions (forebrain cortex, cerebellum, hippocampus) + BDNF gene expression	qRT-PCR (BDNF + synaptic genes) + Western blot (SNAP25, syntaxin-1, PSD-95 etc.) + TEM synaptic ultrastructure + confocal imaging of BDNF in	Low-dose perinatal Pb (Pb-B <10 μg/dL) induced synaptic ultrastructural pathology (swelling, thickened synaptic cleft, vesicle accumulation) + altered key synaptic proteins (SNAP25↓ hippocampus; syntaxin-1↓ cerebellum/hippocampus; PSD-	Not extracted as pooled effect size (group comparisons significant; % changes reported for some proteins)	Synaptic dysfunction via impaired exocytosis machinery + mitochondrial alterations + reduced BDNF retrograde signaling affecting vesicle docking/release	Not assessed (no behavioral tests)	Missing unique Pb developmental synapse/TEM study; excellent mechanistic support Pb→BDNF↓ + synaptic dysfunction

										primary cerebellar neurons	95↓ cortex/cerebellum & ↑ hippocampus) + lower BDNF in all brain structures				

Table S3. Quality assessment of included human observational studies using the Newcastle-Ottawa Scale (NOS)

Study ID	Design	NOS_Selection (0-4)	NOS_Comparability (0-2)	NOS_Outcome/Exposure (0-3)	NOS_Total (0-9)	Quality (Good/Fair/Poor)	Notes
[13]	Human observational (children; BLRV groups)	3	1	2	6	Fair	Good biomarker measurement (Pb + plasma BDNF), but cross-sectional and limited confounder handling details
[26]	Birth cohort (prenatal MeHg; cord serum BDNF)	4	2	2	8	Good	Strong cohort, clear exposure biomarkers (cord blood + hair), robust model; main limits: single-time outcome
[28]	Cross-sectional (chronic As; MMSE + sBDNF)	4	2	2	8	Good	Large sample, multiple exposure biomarkers (water/hair/nails), cognitive outcome MMSE; cross-sectional design limitation

[29]	Cross-sectional (children; BLL + CPMS + BDNF)	3	1	2	6	Fair	Small sample (N=72), good exposure and BDNF mRNA/protein, but limited adjustment + cross-sectional
[27]	Birth cohort (cord Mn; cord BDNF; infant DQ)	4	2	2	8	Good	Prospective cohort + neurodevelopment endpoint + confounder adjustment; strong biologic plausibility (Mn–BDNF–DQ)

Table S4. Quality and reliability assessment of experimental animal and in vitro studies using SciRAP criteria

Study ID	Study type	Exposure characterization	Outcome assessment	Randomization/Blinding	Statistics & reporting	Overall reliability (High/Med/Low)	Notes
[36]	Animal in vivo (neonatal mice)	TRUE	TRUE	Partial	TRUE	Medium	Strong mechanistic design (MMP/BDNF + behavior), but randomization/blinding not clearly reported
[42]	Animal in vivo (rat)	TRUE	TRUE	Partial	TRUE	Medium	Clear groups + Pb biomarker + BDNF/behavior; intervention study; blinding not described
[35]	Animal in vivo (mouse)	TRUE	TRUE	Partial	TRUE	High	Strong exposure quantification + behavior + mechanistic epigenetics/BDNF; includes supportive human mediation (not scored with NOS)
[41]	Animal in vivo (mouse)	TRUE	TRUE	Partial	TRUE	Medium	Dose-response and mechanistic proteins; missing details on randomization/blinding
[34]	Animal in vivo (rat; Ni+Al mixture)	TRUE	TRUE	Partial	TRUE	Medium	Good mixture exposure + BDNF/NGF + behavior; some reporting gaps on blinding/randomization
[45]	Animal in vivo (rat; Cd + thymol)	TRUE	TRUE	Partial	TRUE	Medium	Good behavioral battery + hippocampal BDNF; limited reporting on allocation concealment/blinding

[33]	Animal in vivo (chicken; HgCl2 + Se)	TRUE	TRUE	Partial	TRUE	Medium	Strong mechanistic pathway activation; no behavior; randomization/blinding unclear
[32]	Animal in vivo (rat; MeHg vs IHg)	TRUE	TRUE	Partial	TRUE	High	Excellent multi-omics exposure profiling + BDNF matrices; behavioral endpoints missing but mechanistic design strong
[30]	Animal in vivo (maternal Pb; offspring)	TRUE	TRUE	Partial	TRUE	Medium	Developmental model + brain BDNF/TrkB; confounder control limited; blinding unclear
[31]	Animal in vivo (postnatal Mn; hippocampus)	TRUE	TRUE	Partial	TRUE	Medium	Good mechanistic NMDA–CREB–BDNF pathway; no behavior; allocation/blinding unclear
[39]	Animal in vivo (Pb + HFD)	TRUE	TRUE	Partial	TRUE	High	Strong combined stressor design + behavior + CREB–BDNF + insulin signaling; reporting generally solid
[40]	Animal in vivo (perinatal Pb; synapses TEM)	TRUE	TRUE	Partial	TRUE	High	Very strong mechanistic ultrastructure + synaptic proteins + BDNF; no behavior but excellent exposure/outcome measurement
[38]	In vitro (primary neurons)	TRUE	TRUE	N/A	TRUE	High	High mechanistic certainty (BDNF knockdown/rescue), clean design, clear endpoints
[37]	In vitro (primary hippocampal neurons)	TRUE	TRUE	N/A	TRUE	High	Strong mechanistic Cd→BDNF–TrkB disruption + Zn rescue; detailed cellular endpoints
